# Peri-implant health, clinical outcome and patient-centred outcomes of implant-supported overdentures in the mandible and the maxilla

**DOI:** 10.1038/bdjopen.2017.17

**Published:** 2017-09-08

**Authors:** David Offord, Grant Mathieson, Nicola Kingsford, Carine Matthys, Maarten Glibert, Hugo De Bruyn

**Affiliations:** 1 Vermilion, Edinburgh, UK; 2 Department of Periodontology and Oral Implantology, University of Ghent, University Hospital Ghent, Ghent, Belgium

## Abstract

**Objectives/Aims:**

The primary aim of this retrospective pilot study was to evaluate the clinical outcome of overdentures on four non-splinted maxillary implants compared to the mandible using locator attachments and secondly to assess patient's opinion of the treatment.

**Materials and Methods:**

The treatment protocol used here is summarised as a single-stage surgical approach followed by immediate loading (same day in 12 of 17 patients) of a removable prosthesis in the maxilla and mandible. Most of the implants were installed into fresh extraction sockets. Clinical outcomes were evaluated in 68 southern implants, straight (non co-axis) or angulated (co-axis) in 17 patients. Patients were examined by independent examiners at an average follow-up of 14.5 months after implant placement.

**Results:**

Outcomes measured were implant survival, bone loss, bleeding on probing, probing pocket depths and plaque score in addition to quality of life measured with OHIP-14 questionnaires. An overall implant survival of 100% was achieved. The mean marginal bone level (mm) over the entire cohort of 66 measured implants was (1.4 mm; range, 0–5.5). A significant difference (*P*=0.01) was found between bone level, from implant head to bone contact in the maxilla (M, 0.9 mm; s.d., 1.1; range, 0–4) and the mandible (M, 1.7; s.d., 1.0; range, 0–5.5). The marginal bone-to-implant head distance with the angulated co-axis implants was 1.9 mm (s.d., 1.5; range, 0–5.5) compared to non co-axis, mean 1.2 mm (s.d., 1.1; range, 0–4) (*P*=0.01). The OHIP-14 overall mean was 3.3 (out of a maximum of 56).

**Conclusion:**

The implant survival was 100% and the patients benefited from the overdenture treatment on four non-connected implants. The extremely low OHIP-14 indicated a very high level of patient satisfaction following treatment. The results of this study merit further long-term investigation to fully investigate the success of immediately loading implants in the maxilla as well as cost-benefit.

## Introduction

Dental implants yield an above 95% survival in long-term studies under most treatment indications. Also immediate loading is predictable with single implants or when multiple implants are splinted with a fixed reconstruction in maxilla and mandible.^1^ The clinical outcome of implant-supported overdentures is very successful in the mandible under delayed and immediate loading conditions and two implants are considered the standard of care.^1^

A recent systematic review indicated that the number of studies providing information on overdentures in the maxilla is rather limited.^2^ Machined surface implants yield high failure rates and often four–six implants are suggested to support a splinted reconstruction. Sadowsky and Zitzmann^2^ recently carried out a systematic review of 23 publications relating to 20 cohort studies looking at maxillary implant overdentures. They concluded that four–six implants are widely applied in successful cohort studies and found no distinct evidence that implant splinting with a bar is superior to single attachments in terms of implant survival, in general splinted and solitary anchorage systems are both advocated. Furthermore, technical issues with attachments or soft tissue irritations under bars have been described and difficulty of cleaning has been reported with bar anchorages. The success of non-axially loaded implants as a technique to overcome the problem of bone resorption particularly in the posterior maxilla has been described in some detail.^3^

There is evidence in the dental literature showing successful immediate loading of implant overdentures in the mandible, but there is a paucity of evidence regarding the maxilla. This is mainly related to jeopardised bone condition making immediate loading more problematic in the maxilla, especially in elderly patients. In a systematic review on immediate loading in various treatment indications,^1^ only two prospective clinical studies were available. Eccellente *et al.*^4^ used the Ankylos Syncone system (Dentsply Friadent, Mannheim, Germany) in 45 subjects with four non-splinted implants. A prefabricated conical crown was adapted to the relined existing denture. The conical crown concept resulted in stable complete denture retention, a reduced denture base and facilitated oral hygiene. The overall implant survival rate was 97.8% during an average observation period of 26 months. Pieri *et al.*^5^ attached maxillary overdentures in 22 consecutive patients on four–five implants connected by a bar and this yielded a 97.1% survival after one year. The most common prosthetic complication was frequent relining of the denture in the initial weeks in 27% of the patients. The patient’s subjective appreciation of function and satisfaction increased significantly in comfort, functional and aesthetic parameters. The patients found it difficult to maintain the high level of oral hygiene required. Although it is suggested that treatment outcome may be good, one should realise that current evidence to recommend this treatment on a routine basis is insufficient and comparative studies to provide surgical or prosthetic guidelines related to patient selection are unavailable.

The primary aim of this retrospective pilot study was to evaluate the clinical outcome of overdentures on four non-splinted maxillary implants compared to the mandible using locator attachments (Zest Anchors, Carlsbad, CA, USA) and second to assess patient’s opinion of the treatment.

## Materials and methods

### Patient selection

From 2011 to 2014, 21 either dentate or edentulous patients were treated at Vermilion – The Smile Experts Ltd, Edinburgh with implants in the maxilla and mandible to provide stability for an overdenture. Smokers, diabetics and patients with history of periodontitis were not excluded and there was no restriction based on the opposing dentition. Patients were personally invited by the dental surgeon (DO) by phone or mail to participate in a clinical examination and 17 patients agreed to be checked by independent examiners from Ghent University, Belgium. NHS ethical approval was not required under the terms of the Governance Arrangements for the Research Ethics Committee (South East Scotland Ethics) and the advice was based on the study being limited to utilisation of data previously collected in the course of normal care (reference number NR/1408AB14).

### Surgical phase

**Figure 1:**
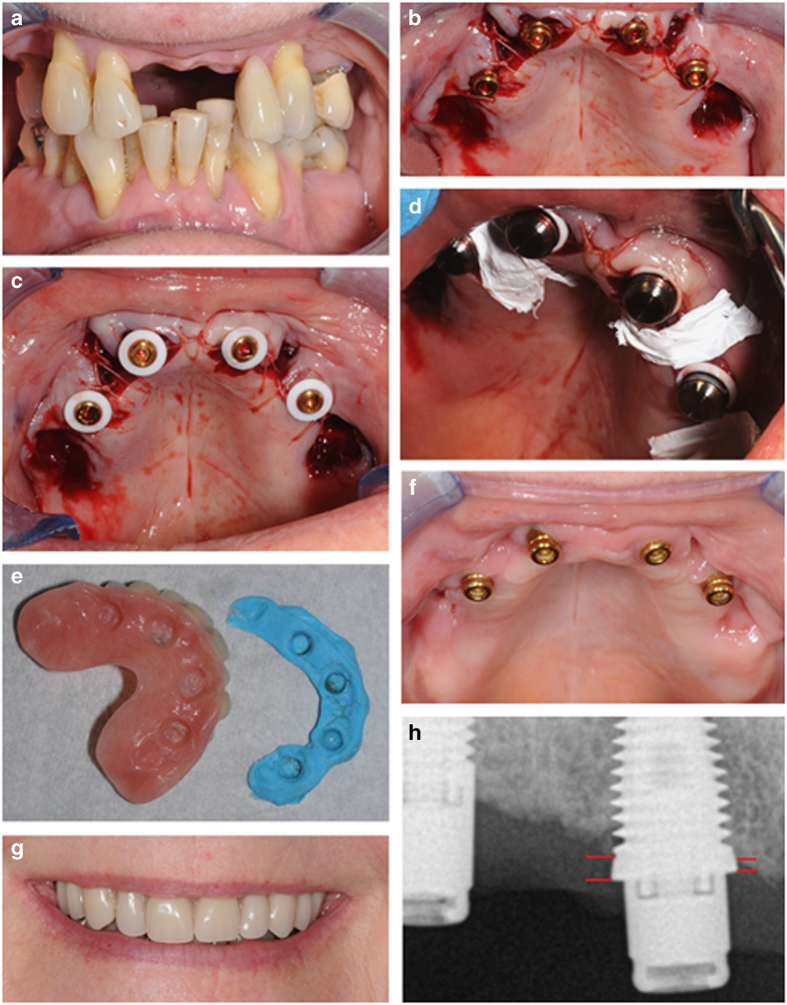
(**a**) A 64-year-old female, with defective maxillary dentition received four immediately placed implants simultaneously with extractions using (**b**) four implants were installed (3×11.5 mm and 1×10 mm) and to enhance parallelism one of the anterior implants was a 12° co-axis, locators (3 mm anterior, 4 mm posterior) were placed and tightened. The flap was sutured around the abutments (**c**). Silicone rings (**d**) and housings were placed and the surgical site was protected with PTFE tape (**e**) prior to functional connection and relining with UFI-GEL denture lining. (**f**) Healing at 6 weeks was uneventful (**g**) and patient was provided with a satisfying result functionally and aesthetically. (**h**) Radiographic image showing an example of how bone level to implant head changes was measured in mm on implant 14. Red lines indicate areas of measurement on the medial and distal side of the implant.

Surgery was carried out by one oral surgeon (DO) by initially raising of flap followed by implant placement following the manufacturer’s recommendations (Figure 1b–d). Implants (Southern Implants Ltd, Irene, South Africa) were either 4 or 5 mm wide and having external Hex or internal connection. They were 10–13 mm long and either straight (non co-axis) or with a built-in angulated platform of 12° or 24° (co-axis). Implants were made from Grade 4 cp Ti surface roughened by alumina blasting and chemical cleaning. Implants were inserted with a 25–40 Ncm initial torque force to ensure proper initial stability. All abutments were locator-type self-aligning stud abutments with cuff heights of 3, 4 or 5 mm depending on the soft tissue thickness.

After implant installation, immediate placement of locator abutments was carried out with immediate loading of the interim prosthesis (same day) whenever implant insertion torque exceeded 25 Ncm (Figure 1e,f). After suture of the flap around the abutments, silicon rings and housings were placed and the surgical site protected with PFTE tape prior to functional connection and relining with UFI-GEL (Voco Gmbh, Cuxhaven, Germany) denture lining. The temporary acrylic prosthesis bridge is then put into place and UFI-GEL sets to pick up the locator\housings and put in position. Patients who had an immediately loaded interim prosthesis were given homecare and soft diet instructions and healing was checked after 2 weeks. Patients were asked not to remove the prosthesis until the 2-week check-up. At the 2-, 4- and 6-week check-up, the prosthesis was removed and cleaned by the prosthodontist (GM). When implants were delayed loaded, the definitive removable prosthesis was installed after 6 months. The implants were monitored at 2, 4, 6 weeks, and 6 months post placement. A cone beam CT scan was taken pretreatment for implants placed in the maxilla. Peri-apical radiographs and clinical photographs were taken (Figure 1h).

### Clinical evaluation

Clinical evaluation was carried out at a mean follow-up of 14.5 months after implant placement. Bleeding on probing and plaque was measured on four sites (mesial, distal, buccal and lingual) using a dichotomous score (0=no bleeding/no plaque; 1=bleeding/presence of visible plaque) leading to an implant score between 0 and 4. Probing depth was registered on the four sites with a periodontal probe. Clinical implant stability was examined clinically and retention of the prosthesis was checked by rocking the prosthesis and clinical inspection of damaged matrices or fractures.

### Radiographic evaluation

Peri-implant bone levels were measured on radiographs taken at a mean follow-up of 14.5 months after implant placement with the Nomad Pro* handheld X-ray system for intraoral radiographic imaging (1–4 exposures of 60 kV, 2.5 mA for 0.13 s). Digital peri-apical radiographs were taken for each individual implant using the parallel long-cone technique in order to visualise the implant threads and marginal bone-to-implant contact level using the implant–abutment interface as reference point (Figure 1h). A guiding system (Schick, New York, NY, USA) was used to obtain the X-ray direction perpendicular to the sensor and parallel to the implant. Whenever the implant threads were unclear, another radiograph was taken until the bone value could be determined.

### Patient’s opinion questionnaire

The patients filled out the Oral Health Impact Profile (OHIP-14) questionnaire at a mean follow-up of 14.5 months after implant placement to evaluate the impact of the overdenture treatment on oral health-related quality of life by focusing on eating and speaking comfort as well as physical, psychological and social discomfort.^6–8^ The index measures the people’s perception of the social impact of oral disorders on their well-being on a 5-point scale ranging from very negative (score 4) to very positive (score 0). The average score per question as well as the total score of 14 items was calculated

### Data analysis

The mesial and distal bone was measured around each implant, and a Wilcoxon signed-rank test was applied to test the differences between the mesial and distal bone levels. Because these were not statistically significantly different, the mesial and distal values were averaged to obtain one value per implant.

Mann–Whitney *U*-tests were used to analyse differences in marginal bone level changes based on location (mandible versus maxilla), angulation (angulated implants versus straight implants and smoking behaviour (smokers versus non-smokers). All statistical analyses were performed using SPSS 22.0 (IBM SPSS Statistics for Windows, Armonk, NY: IBM Corp.) with a pre-set significance level of *P*⩽0.05.

## Results

Data were included from 21 consecutively treated patients, 17 patients agreed to participate (5 males and 12 females, mean age 65.1 years; s.d., 9.0; range, 48–79). The mean follow-up time was 14.5 months (range, 1–36; s.d., 11.5). In total, 68 implants were examined and clinically inspected. Twenty-six implants in nine patients were placed in the mandible and 42 in 11 patients were placed in the maxilla; 45 were straight and 21 co-axis. Three patients indicated they were smokers and 14 were non-smokers. All clinically examined implants were immobile without signs of pain and all supporting a functional overdenture. This trial showed good implant survival without any failures. The average probing depth was 2.5 mm (s.d., 0.8; range, 1.3–5.3) with only three patients showing probing depths greater than 3 mm. The mean plaque index was 20% (s.d., 34%; range, 0–10) and the mean bleeding index was 0.21 (s.d., 0.3; range, 0–1).

Analysed data included bone loss related to 66 implants. Albrektsson *et al.*^9^ described an acceptable bone loss of 1.5 mm during the first 12 months and a further maximum of 0.2 mm yearly. A total of six implants in two patients in this study lay outside this acceptable limit. The first of these patients prior to implant placement had aggressive periodontal disease, was a smoker, a bruxist and had an atrophic maxilla lacking height and width. The other patient’s implants were placed in the edentulous mandible; she had a periodontal disease history and was also a past smoker.

**Figure 2:**
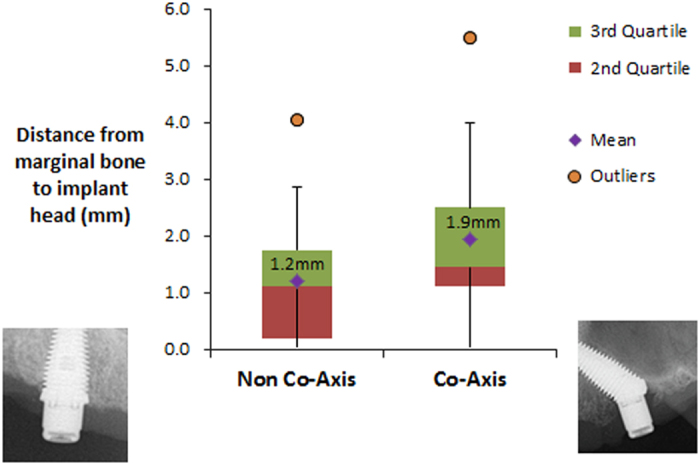
Distance from marginal bone to implant head (mean±s.d. mm) in non co-axis and co-axis implants.

A significant difference (*P*=0.01) was found between bone level, from implant head to bone contact in the maxilla (M, 0.9 mm; s.d., 1.1; range, 0–4) and the mandible (M, 1.7; s.d., 1.0; range, 0–5.5). The marginal bone to implant head distance with the angulated 12° or 24°, co-axis implants were 1.9 mm (s.d., 1.5; range, 0–5.5) as compared to the non co-axis, mean 1.2 mm (s.d., 1.1; range, 0–4) (Figure 2). These results were significantly different (*P*=0.01). The bone loss was measured on a total of 13 implants measured in smokers (mean, 1.8 mm; s.d., 1.2; range, 0–4) and 53 implants from non-smokers (mean, 1.3 mm; s.d., 1.1; range, 0–5.5). Although the difference in bone loss was not significantly different between the groups (*P*=0.15), there is also trend towards more expressed bone loss in the smoking group.

Patients completed the OHIP-14 questionnaire only at the time of the final clinical examination. An extremely low overall mean of 3.6 (s.d., 3.6; range, 0–11) from a possible maximum of 56 OHIP-14 points indicating a high quality of life after treatment (Table 1).

## Discussion

This is a retrospective study describing an overdenture treatment protocol that can be summarised as a single-stage surgical approach followed by immediate loading (same day in 12 of the 17 patients) of a removable prosthesis. Most of the implants were installed into fresh extraction sockets. Our aim here was to reduce the amount of surgery and the restoration placement without compromising the success of the implant. An overall implant survival of 100% was achieved, which is in line with similar reports on immediate loading in fully edentulous maxillae and mandibles.^1^ This clinical outcome on implant survival is in line with other reports in the literature.^4,5,10^ There has become an increasing amount of evidence that the modern implant design do not yield a high failure rate when implants are placed into fresh sockets and then immediately loaded.^1^ There have been some comprehensive systematic reviews of early or immediate loading of mandibular overdentures compared to delayed loading.^11,12^ These analyses concluded there was no significant difference between the two protocols with a loading period of up to 2 years. Another systematic review based on 25 studies reported bone loss of 0–0.2 mm in early loading studies and 0.7 mm in immediate loading studies.^1^ This difference has been explained by the time the baseline radiographs are taken. In immediately loaded implants, the baseline is taken at the time of surgery and not after a period of prolonged healing.

In this study, the mean marginal bone level (mm) over the entire cohort of 66 measured implants was (1.4 mm; range, 0–5.5)). The acceptable bone loss according to the success criteria, described by internationally well acclaimed Albrektsson *et al.*,^5^ is 1.5 mm during the first year and then a stabilizing of crestal bone loss to only 0.2 mm yearly. Our data show this apart from one patient whose bone loss in all four implants was over 2.5 mm and up to 5.5 mm. This patient before placement of implants had aggressive periodontal disease, was smoker, a bruxist and had an atrophic maxilla. There were no significant differences between the maxillary and mandibular bone levels. Also the co-axis (angulated) implants did not seem to result in differences in peri-implant bone level compared to straight implants. Other studies have also shown no difference in bone loss between straight or tilted implants.^13–15^

Very few studies have evaluated patient satisfaction with implant treatment. These two papers by Attard *et al.*^16,17^ focussed on implants in the mandible and measured patient mediated and economic factors following immediate loading of implants with mandibular overdentures. In the current study, we measured the patient satisfaction with treatment using the OHIP-14. The overall mean of 3.3 (out of a maximum of 56) was extremely low indicating a very high level of patient satisfaction following treatment. This study contained no baseline measurement for the OHIP-14 questionnaire, this could be attributed to the immediate loading protocol known to yield high satisfaction compared to delayed loading.^18^

It is not known what the implications of this procedure over the long term are going to be. Dental implants are known to function well, but an ongoing study of this cohort over time will show us if this procedure has an impact on the success of implants and the prosthesis in the long term.^1^ The patients included in this study follow regular maintenance of their implants and prostheses with adjustments made regularly, this may overcome some of the biological and technical issues that may occur when cases are not followed up regularly.

We realise we have a limited number of cases here because this is a working daily practice. However, the study can continue with honest reporting on all issues over time to get a gauge on the success of this procedure particularly in the maxilla. There was one notable aspect in regard to the OHIP-14 results in every patient group, the scores are very low, indicating a good quality of life. Because we had over 80% (17/21) patients willing to come back and participate in the study, there may be a bias in the patient satisfaction (OHIP-14) results.

Another limitation of this retrospective study was no baseline measurements taken for OHIP-14 or radiographs for bone measurement. The only radiographs available were taken as routine radiographs and were not accurate or clear enough to carry out bone measurements. In prospective studies, the authors will be taking baseline radiographs from the day when the trans-mucosal part pierces the mucosal tissue and annually thereafter.

## Conclusion

The implant survival was 100% and the patients benefited from the overdenture treatment on four non-connected implants. There is limited long-term data on procedural recommendations for the minimum number of implants required, length and the success of non-splinted immediately loaded prosthesis in the maxilla. The results of this study merit further long-term investigation to fully investigate the success of immediately loading implants in the maxilla as well as cost-benefit.

## Figures and Tables

**Table 1:** OHIP-14 questionaire

Questions 1–14 (score range, 0–4)		Mean±s.d. (range, 0–4)
1	Have you had trouble pronouncing any words because of problems with your teeth, mouth or dentures?	0.5±0.7 (0–2)
2	Have you felt your sense of taste has worsened because of problems with your teeth, mouth or dentures?	0 (0)
3	Have you had painful aching in your mouth	0.5±0.7 (0–2)
4	Have you found it uncomfortable to eat any foods because of problems with your teeth, mouth or dentures?	0.8±1.0 (0–3)
5	Have you been self-conscious because of your teeth, mouth or dentures?	0.2±0.4 (0–1)
6	Have you felt tense because of problems with your teeth, mouth or dentures?	0.2±0.4 (0–1)
7	Has your diet been unsatisfactory because of problems with your teeth, mouth or dentures?	0.3±0.4 (0–2)
8	Have you had to interrupt meals because of problems with your teeth, mouth or dentures?	0.5±0.7 (0–2)
9	Have you found it difficult to relax because of problems with your teeth, mouth or dentures?	0.2±0.4 (0–1)
10	Have you been a bit embarrassed because of problems with your teeth, mouth or dentures?	0.2±0.4 (0–1)
11	Have you been a bit irritable with other people because of problems with your teeth, mouth or dentures?	0.1±0.2 (0–1)
12	Have you had difficulty doing your usual jobs because of problems with your teeth, mouth or dentures?	0.1±0.2 (0–1)
13	Have you felt that life in general was less satisfying because of problems with your teeth, mouth or dentures?	0.1±0.2 (0–1)
14	Have you been totally unable to function because of problems with your teeth, mouth or dentures?	0 (0)
	Total	3.6±3.6 (0–11)

A series of 14 questions assessing the patient’s quality of life indicators related to oral health conditions. Scale ranges from positive (0=Never) to negative (4=Always).

## References

[bib1] De Bruyn H, Raes S, Ostman PO, Cosyn J. Immediate loading in partially and completely edentulous jaws: a review of the literature with clinical guidelines. Periodontol 2000 2014; 66(Suppl 1): 153–187. 25123767 10.1111/prd.12040

[bib2] Sadowsky SJ, Zitzmann NU. Protocols for the maxillary implant overdenture: a systematic review. Int J Oral Maxillofac Implants 2016; 31(Suppl s): 182–191. 10.11607/jomi.16suppl.g5.2 27228249

[bib3] Krekmanov L, Kahn M, Rangert B, Lindstrom H. Tilting of posterior mandibular and maxillary implants for improved prosthesis support. Int J Oral Maxiillofac Implants 2000; 15: 405–414. 10874806

[bib4] Eccellente T, Piombino M, Piattelli A, D'Alimonte E, Perrotti V, Iezzi G. Immediate loading of dental implants in the edentulous maxilla. Quintessence Int 2011; 42(Suppl 4): 281–289. 21516273

[bib5] Pieri F, Aldini NN, Fini M, Marchetti C, Corinaldesi G. Immediate functional loading of dental implants supporting a bar-retained maxillary overdenture: preliminary 12-month results. J Periodontol 2009; 80(Suppl 11): 1883–1893. 19905959 10.1902/jop.2009.090227

[bib6] De Bruyn H, Raes S, Matthys C, Cosyn J. The current use of patient-centered/reported outcomes in implant dentistry: a systematic review. Clin Oral Implants Res 2015; 26(Suppl 11): 45–56. 26385620 10.1111/clr.12634

[bib7] Roumani TI, Oulis CJ, Papagiannopoulou V, Yfantopoulos J. Validation of a Greek version of the oral health impact profile (OHIP-14) in adolescents. Eur Arch Paediatr Dent 2010; 11(Suppl 5): 247–252. 20932400 10.1007/BF03262756

[bib8] Slade GD. Derivation and validation of a short-form oral health impact profile. Community Dent Oral Epidemiol 1997; 25(Suppl 4): 284–290. 9332805 10.1111/j.1600-0528.1997.tb00941.x

[bib9] Albrektsson T, Zarb G, Worthington P, Eriksson AR. The long-term efficacy of currently used dental implants: a review and proposed criteria of success. Int J Oral Maxillofac Implants 1986; 1(Suppl 1): 11–25. 3527955

[bib10] Agliardi E, Panigatti S, Clericò M, Villa C, Malò P. Immediate rehabilitation of the edentulous jaws with full fixed prostheses supported by four implants: interim results of a single cohort prospective study. Clin Oral Implants Res 2010; 21(Suppl 5): 459–465. 20105197 10.1111/j.1600-0501.2009.01852.x

[bib11] Alsabeeha N, Atieh M, Payne AG. Loading protocols for mandibular implant overdentures: a systematic review with meta-analysis. Clin Implant Dent Relat Res 2010; 12(Suppl 1): e28–e38. 19438962 10.1111/j.1708-8208.2009.00152.x

[bib12] Ma SI, Payne AG. Marginal bone loss with mandibular two-implant overdentures using different loading protocols: a systematic literature review. Int J Prosthodont 2010; 23(Suppl 2): 117–126. 20305848

[bib13] Agliardi E, Clericò M, Ciancio P, Massironi D. Immediate loading of full-arch fixed prostheses supported by axial and tilted implants for the treatment of edentulous atrophic mandibles. Quintessence Int 2010; 41(Suppl 4): 285–293. 20305862

[bib14] Koutouzis T, Wennström JL. Bone level changes at axial- and non-axial-positioned implants supporting fixed partial dentures. A 5-year retrospective longitudinal study. Clin Oral Implants Res 2007; 18(Suppl 5): 585–590. 17608740 10.1111/j.1600-0501.2007.01386.x

[bib15] Aparicio C, Perales P, Rangert B. Tilted implants as an alternative to maxillary sinus grafting: a clinical, radiologic, and periotest study. Clin Implant Dent Relat Res 2001; 3(Suppl 1): 39–49. 11441542 10.1111/j.1708-8208.2001.tb00127.x

[bib16] Attard NJ, David LA, Zarb GA. Immediate loading of implants with mandibular overdentures: one-year clinical results of a prospective study. Int J Prosthodont 2005; 18(Suppl 6): 463–470. 16335163

[bib17] Attard NJ, Laporte A, Locker D, Zarb GA. A prospective study on immediate loading of implants with mandibular overdentures: patient-mediated and economic outcomes. Int J Prosthodont 2006; 19(Suppl 1): 67–73. 16479763

[bib18] Fischer K, Stenberg T. Three-year data from a randomized, controlled study of early loading of single-stage dental implants supporting maxillary full-arch prostheses. Int J Oral Maxillofac Implants 2006; 21(Suppl 2): 245–252. 16634495

